# A computational approach for genome-wide mapping of splicing factor binding sites

**DOI:** 10.1186/gb-2009-10-3-r30

**Published:** 2009-03-18

**Authors:** Martin Akerman, Hilda David-Eden, Ron Y Pinter, Yael Mandel-Gutfreund

**Affiliations:** 1 Department of Biology, the Technion - Israel Institute of Technology, Haifa 32000, Israel; 2 Department of Computer Science, Technion - Israel Institute of Technology, Haifa 32000, Israel

## Abstract

Alternative splicing is regulated by splicing factors that serve as positive or negative effectors, interacting with regulatory elements along exons and introns. Here we present a novel computational method for genome-wide mapping of splicing factor binding sites that considers both the genomic environment and the evolutionary conservation of the regulatory elements. The method was applied to study the regulation of different alternative splicing events, uncovering an interesting network of interactions among splicing factors.

## Abstract 2

A computational method is presented for genome-wide mapping of splicing factor binding sites that considers both the genomic environment and evolutionary conservation.

## Background

Alternative splicing (AS) is a post-transcriptional process responsible for producing distinct protein isoforms as well as down-regulation of translation. Many experimental and computational studies revealed that AS can be regulated in a tissue-specific manner [1-4] during embryonic development [5] or in response to particular cellular stimuli [6]. AS regulation is known to be mediated by many splicing factors (SFs), generally belonging to the serine-arginine-rich (SR) and heterogeneous nuclear ribonucleoprotein (hnRNP) families [7]. These SFs can instigate positive or negative effects on the splicing reaction by differentially interacting with exonic or intronic splicing enhancers and silencers.

SFs tend to assemble into a large complex known as the spliceosome [8]. Despite their remarkable diversity, SFs share common characteristics. Several SFs, such as the polypyrimidine tract-binding protein (PTB) [9] and hnRNP A1 [10], bind the pre-mRNA in multimeric units. In several cases the binding sites are found in relatively long RNA stretches, such as the polypyrimidine tract that harbors binding sites for PTB and CELF proteins [11], the poly U sequences (length 5-10 nucleotides) that bind the TIA1/TIAL1 proteins [12], and G-rich sequences (between one to several G triplets) that have been shown to bind the hnRNP H/F [13]. Another example is the NOVA-1 splicing factor, which was reported to bind clusters of YCAY sequences that are specifically located nearby the splice sites of alternatively spliced exons [14]. The preference of some of the SFs to bind consecutive elements can partially be explained by the modularity of their structure, usually possessing several RNA recognition motifs (RRMs), which are involved in RNA binding [15].

As is true with many regulatory sequences, splicing regulatory elements tend to be conserved among species [16]. These results are consistent with the overall high evolutionary conservation levels observed in AS-related introns [17,18] and in the codon wobble position of alternative exons [19]. Furthermore, high evolutionary conservation has been associated with constitutive splicing. In a recent study, Voelker and co-authors [20] identified sequence motifs that resemble *cis*-regulatory binding sites and that were found to be conserved in constitutive exons of six eutherian mammals. Unexpectedly high evolutionary conservation was also observed in upstream distal splice sites in tandem acceptors that are constitutively spliced [21]. Clustering of evolutionarily conserved *cis*-regulatory elements has been previously demonstrated for transcription factors binding sites. Recent transcription factors binding site prediction tools have demonstrated that consideration of neighboring effects dramatically improves prediction performance compared to strategies that consider only a single site [22-25].

In recent years, several methodologies for identifying splicing factor binding sites (SFBSs) have been developed [19,26-29]. Generally, these methods employ two major approaches: statistical methods based on overabundance of motifs in regulatory regions (for example, [27]); and methods that are based on identifying motifs from experimental binding data (for example, [26]); for a review, see [30]. Several statistical approaches for searching splicing regulatory motifs, such as that of Goren *et al*. [19], have also considered evolution conservation. Overall, the available methods concentrate on the core binding motif and do not consider genomic information from flanking regions. Here we present a novel computational approach for predicting and mapping SFBSs of known splicing factors that considers both the genomic environment as well as the evolutionary conservation of the splicing factor *cis*-regulatory elements. The method was trained and tested on experimentally validated sequences, displaying high accuracy of 93% with a relatively low false positive rate of 1% on the tested data. In addition, the method was applied to different sets of exons and introns, and detected an enrichment of SFBSs in different types of AS, such as cassette exons (CEs), alternative donors (ADs), and alternative acceptors (AAs), compared to constitutive exons. Furthermore, we used our method to study splicing regulatory circuits connecting the subset of splicing factors that were available in our dataset. Careful analysis of the splicing network's structure revealed distinct features, characteristic of other regulatory networks, such as transcription networks. Specifically, we identified clear differences between tissue-specific versus broadly expressed SFs.

## Results and discussion

### A method for mapping splicing factor binding sites

**Figure 1:**
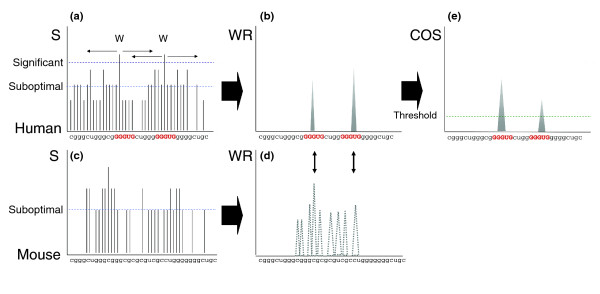
Schematic representation of the COS(WR) function. **(a) **A candidate human sequence is queried with a regulatory motif. **(b) **The weighted rank (WR) is computed only for significant positions by combining all scores above the suboptimal threshold in a sequence window of size *w*. **(c, d) **We calculate WR scores for the candidate's homologous region in mouse that aligns to the human sequence flanking the significant hits. **(e) **WR scores of the candidate sequence and its homologue are combined by calculating the Conservation Of Score (COS).

During the splicing process, many SFs bind and detach from the pre-mRNA at both the exonic and intronic sequences flanking the splice sites. To accommodate for such dynamic interactions, most SFs bind short (4-10 nucleotide) and degenerate sequences (Table S1 in Additional data file 1) [11,14,26,31-53]. As a result, SFBSs are difficult to predict based on motif profiles alone. In order to improve SFBS prediction, we sought to consider sequence information derived from their genomic context as well as evolutionary information. The rationale behind our method relies on two main assumptions: sequence signals flanking a binding motif are informative for binding site recognition; and binding sites tend to be evolutionarily conserved. A diagram of the procedure is illustrated in Figure 1.

### Multiplicity score

As a first step to identify SFBSs, we search a target sequence for a match to a known binding motif. For this purpose a binding motif is represented as a consensus sequence, using the IUPAC definition. The list of binding motifs used in this study to test the algorithm is given in Table S1 in Additional data file 1. The list was generated from the literature as described in the Materials and methods section and it includes only motifs that were experimentally verified (see references in Table 1). Subsequently, each sequence was scored for a match, as described in detail in the Materials and methods section. Upon identifying a significant match to a single motif (*S*^*sig*^; see Materials and methods), we extended our search to a sequence window of size *w *flanking *S*^*sig*^, searching for other short sequences that resemble the sequence of the query motif. Our assumption was that weak signals around the protein binding sites may aid in attracting the SFs to their binding sites, which are generally of low sequence specificity [54]. In addition, though it is not general to all SFs, some splicing regulatory proteins such as NOVA-1 [14] tend to bind to clusters of short binding motifs. In order to account for lower scored hits around a significant hit, we defined a threshold for suboptimal (*S*^*sub*^) hits (see Materials and methods). We then calculated a multiplicity score for the whole window by combining all *S*^*sig *^and *S*^*sub *^within *w *(Figure 1a). The window size was chosen in the training procedure, described below (Table S2 in Additional data file 1). The multiplicity score was computed using a weighted rank (WR) estimation approach (Figure 1b), described in Equation 1. The WR approach was applied here in an attempt to boost the contribution of the high-scored hits within the window (presumably the real binding sites) while lowering the noise from suboptimal (that is, lower affinity sites) and non-significant hits:

(1) $$WR_{w,a}=\sum_{r=1}^{\left|w\right|}a^{-r}S_{r}$$

- where *S*_1 _≥ *S*_2 _≥ ... ≥ *S*_|*w*|_.

*WR*_*w*,*a *_corresponds to the sum of *S*^*sig *^and *S*^*sub *^values decreasingly ranked and divided by the *r*^*th *^power of *a*, where *r *is the position of the value in the ranked list and *a *is chosen to be a small integer (for example, 2).

**Table 1:** Splicing network topological properties

	D	C	L
Splicing network	3	0.31	1.57
ER graphs	6.31 ± 1.34	0.23 ± 0.07	2.68 ± 0.39
Z-score	-2.470	1.097	-2.877
*P*-value (one tail)	0.0068	0.1363	0.002

Comparison between the splicing network properties and 1,000 Erdös-Rényi (ER) random graphs. C, clustering coefficient; D, diameter; L, average length of shortest paths.

### Conservation of score

Calculating the conservation of short *cis*-regulatory elements is not trivial, since in most cases the sequence specificity of a given SF is not limited to a unique arrangement of nucleotides but rather to a group of similar *k*-mers. In addition, positional variations between homologous *cis*-regulatory elements can exist, and still keep their functionality [19,55]. Therefore, in order to calculate the evolutionary conservation between two clusters of *cis*-regulatory elements and still relax the positional and compositional dependencies between homologous sequences, we defined a scoring function called 'Conservation Of Score' (COS; Equation 2), which weights the WR of the target sequence by the difference between itself and the WR of the homologous sequence (*WR*_*w*,*a *_^hom^; Figure 1c-e). Thus, when both *WR*_*w*,*a *_and *WR*_*w*,*a *_^hom ^are similar (that is, the window is conserved) COS increases. In this study we used the human and mouse as primary and homologous sequences, respectively, as in Equation 2:

(2) $$\text{COS(WR)}=WR_{w,a}\cdot (1-\frac{\left|WR_{w,a}-WR_{w,a}^{\mathrm{hom}}\right|}{\max(WR_{w,a},WR_{w,a}^{\mathrm{hom}})})$$

Lastly, in order to separate significant from borderline predictions, we determined a threshold for the COS(WR) values (Figure 1e). This threshold corresponds to the median of the non-zero scores obtained by screening every query against the background model, derived for exons and introns separately (for more details see Materials and methods).

### Evaluating the COS function on known binding sites

In order to provide evidence that the choice of the COS(WR) improves prediction sensitivity, we compared the performance of WR and other estimators - the median (M; Equation 3), the weighted average (WA; Equation 4), and the sum of scores (SS; Equation 5) - to the prediction sensitivity, which was calculated based on a Single Score *S *(Equation 7 in Materials and methods). All estimators were tested with and without the COS function.

(3) *M*_*w *_= *median*{*S*_*i*_|*S*_*i*_, *i *= 1,..., *w*}

(4) $$WA_{w}=\frac{\sum_{i=1}^{\left|w\right|}S_{i}^{2}}{\sum_{i=1}^{\left|w\right|}S_{i}}$$

(5) $$SS_{w}=\sum_{i=1}^{\left|w\right|}S_{i}$$

For this purpose we used a training set that included 56 positive and 502 control sequences (see Materials and methods). The training was conducted as follows: first, scores of 'known SF binding sites' were drawn from the positive set; second, scores for 'non-binding sites' were drawn from a randomly selected set of sequences of equal size from the control set; third, positive and negative scores were ranked together in descending order; and fourth, the true positive rate (TPR) was calculated by splitting the list at the position where the false positive rate reached 1%.

**Figure 2:**
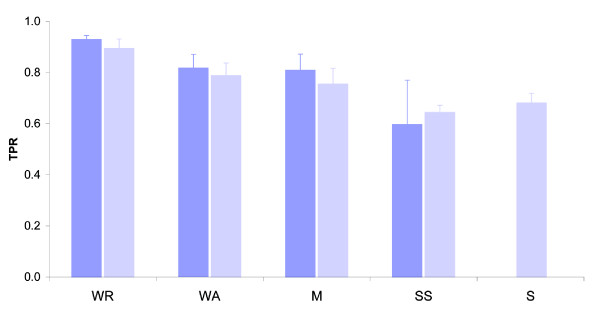
Sensitivity of multiplicity estimators. The average true positive rate (TPR) at a fixed false positive rate of 0.01 when training the data with four different multiplicity estimators: weighted rank (WR), weighted average (WA), median (M) and sum of scores (SS), compared to Single Scores (S). For each estimator the TPR was calculated when considering (dark columns) or not considering (light columns) the Conservation Of Score (COS).

Figure 2 summarizes the average TPRs for ten training iterations (each time selecting randomly an equal number of negative examples from the control set). As shown, the highest scores were achieved when applying the COS(WR) function (TPR = 0.93 ± 0.02), compared to considering a single match *S *(TPR = 0.68 ± 0.04). Other estimators, such as the SS, M, and WA, presented TPRs around 0.6-0.8. These results clearly demonstrate that incorporating information of additional hits around a match outperforms a score based on a single hit. Nevertheless, the best results were achieved when the information from multiple hits within the window was added in a weighted manner, namely the WR approach, where the strong hits are weighted higher and the weak hits are given lower weight. This is likely due to the fact that the most substantial contribution to SF binding in regulatory regions comes from highly significant hits (which could be a single binding site or several consecutive binding sites). However, by themselves these hits may not be sufficient to distinguish true binding sites from background. To further verify that the results are not biased by the relatively small number of sequences in the positive and control set, we applied a similar procedure using the full testing data set (56 positives against 502 negatives). As illustrated in Figure S1 in Additional data file 2, there was no noticeable change in the testing results when including the full dataset. It is important to note that all the training experiments described above were carried out using a predefined set of parameters that were empirically selected using the COS(WR) function, under variable conditions (Table S2 in Additional data file 1). The optimal set of parameters was: *cutoff*^*sig *^at a *P*-value of < 0.01, *cutoff*^*sub *^at a *P*-value of < 0.025, *w *= 50, and *a *= 2. Although these were found as optimal parameters, we observe that using a window size between 30-60 nucleotides produces very similar results when the *cutoff*^*sub *^was changed to a *P*-value of < 0.05 instead of a *P*-value of < 0.025 (results shown in Table S2 in Additional data file 1).

As observed in Figure 2, considering the evolutionary conservation of the scores (using the COS function) improves the prediction's sensitivity, though not dramatically. Further, we wanted to ensure that the high performance of the COS functions is not simply due to the overall higher conservation of the intronic sequences flanking alternative exons relative to the background model [17,18]. Since the high conservation of these regions is related to the SFBSs that are embedded within these sequences, it is practically impossible to tease out the contribution of each feature independently. Nevertheless, to ensure that the overall high conservation does not produce artificial results, we tested whether the COS function would detect other functional motifs, such as transcription binding sites or untranslated region (UTR) motifs, which are not expected to be found within these regions. For that we selected the ten most significant human promoter motifs and ten UTR motifs from Xie *et al*. [56] and tested whether these motifs are detected within our training set by applying the COS(WR) function. As shown in Table S2 in Additional data file 1, the average TPR obtained for both the promoter and UTR motifs was approximately 0.5, what would be expected from a random search. These latter results reinforce the claim that the COS(WR) function specifically improves the detection of true SFBSs within exonic and intronic regions flanking alternative splice sites. It is important to emphasize, however, that the experimental set of data on which the COS(WR) function was originally tested was limited to the available data in the literature, which has been extensively studied and may be biased towards dense and conserved SFBSs.

### Specificity testing on experimentally verified binding sites

**Figure 3:**
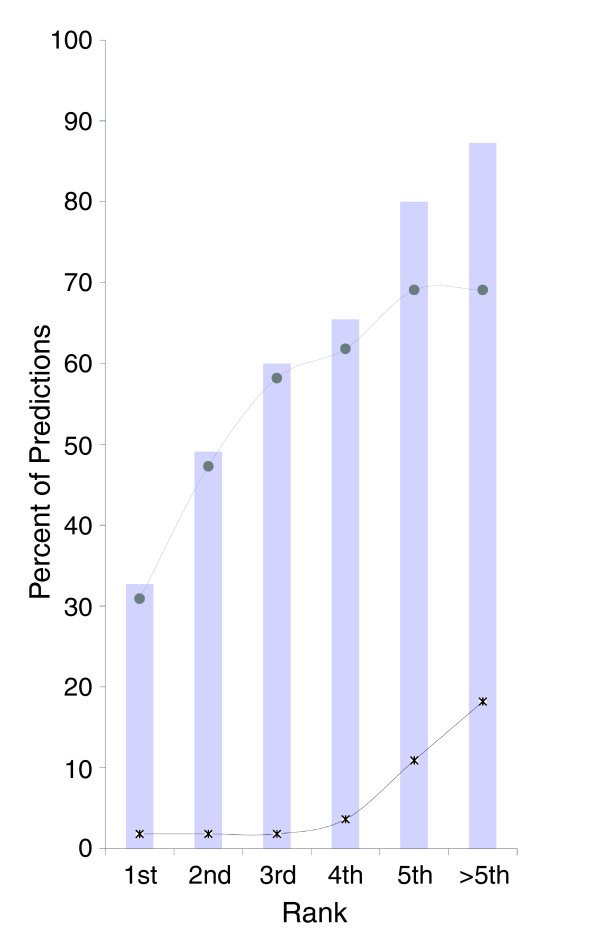
Specificity calculated by the COS(WR) method. The percent of accurate predictions derived from a screening of experimentally validated sequences with 30 different SFBS queries. The x-axis shows the rank of the true positive hits (that is, experimentally validated SFBSs) among the list of predictions derived from the screening. The top curve displays the percent of predictions higher than the COS(WR) threshold and the bottom curve shows the percent of predictions below the threshold.

In order to evaluate the specificity of our method, we measured its ability to predict experimentally verified binding sites of a known SF amongst all other 19 possible SFs. For this purpose we screened a set of core binding sites from experimentally confirmed SFBSs (Additional data file 3) against 30 motifs corresponding to 20 SFs (Table S1 in Additional data file 1). For every core binding site the resulting scores were ranked; ties were given the same ranking index. In cases where the literature reports more than one possible motif for a given SF, we report the highest ranked result. Figure 3 displays the percent of correct predictions amongst the top ranked scores. As shown, for more than 30% of the predictions the highest scored hit (that is, the best prediction) was the 'known binding site' reported in the literature; for almost 60% of the samples the experimentally verified SF was amongst the three best predictions, and in more than 80% of the cases it was amongst the five best predictions. It is important to note that in many cases the core binding site is not clearly defined; therefore, one would expect to find additional SFs in a regulatory sequence that have not been reported in the literature. Moreover, misprediction of some SFBSs could arise from the lack of representation of other sites in the motif set (that is, some motif sets contain only one known SFBS). Nevertheless, when applying the thresholds to the COS(WR) values (described in Materials and methods) we observed that the vast majority of the predictions that were ranked 5 and higher fell above the threshold, while predictions at position 6 or below fell under the threshold (Figure 3).

**Figure 4:**
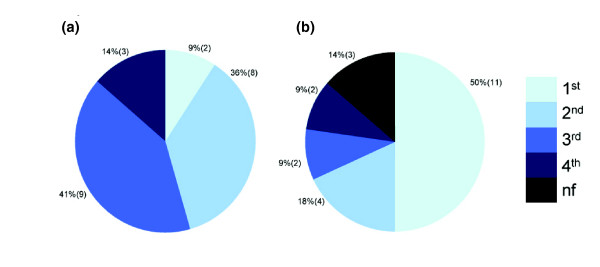
Specificity of the COS(WR) algorithm compared to ESEfinder. A pie chart representing prediction results for four SFs - SF2/ASF, SRp40, SRp55, and SC35 - obtained from screening experimentally validated sequences using **(a) **ESEfinder and **(b) **COS(WR). The different slices represent the percent of true SFBS predictions in the first, second, third, and fourth ranks (color scale is shown on the right). As shown, using the COS(WR) approach, 50% of predictions were ranked at the top rank, while only 9% were top ranked using ESEfinder. nf, not found.

Since in large scale genomic analyses SFBS predictions are expected to be performed on long sequences without previous knowledge of the exact position of the SFBSs, we performed an additional test including both the core and flanking sequences (see Materials and methods). In order to be able to compare our results to another SFBS predictor, we tested the method on four SFs - SF2/ASF, SC35, SRp40, and SRp55 - for which we could apply the well-established predictor ESEfinder [26,57]. Overall, the data included 22 known binding sites and their flanking sequences (total size 100 nucleotides). As shown in Figure 4, our method predicted 50% of the real SFBSs as the first ranked score, whereas ESEfinder predicted only 9% as first ranked scores. It is important to note that the results obtained by our method were applied after optimizing the COS function parameters to our training data (for example, window size, threshold, and so on). Since the optimization applied to our method could not be applied to ESEfinder, the comparison may not be complete.

Taken together, these results demonstrate that the COS(WR) predictor is capable of identifying functional SFBSs with a relatively high level of specificity. Additionally, in comparison to other available tools, the scores derived by the COS(WR) function for different SFBSs are comparable to each other and, thus, they can be ranked in a meaningful way.

### Validating the algorithm against an independent large scale genome analysis

In the last few years, several high throughput genome analyses have been applied to elucidate the targets of different SFs [14,46]. To test the validly of the COS(WR) to detect SF binding signals at the genomic scale, we applied the COS(WR) algorithm to two independent data sets of endogenous target sequences of two different splicing factors, NOVA-1 and SF2/ASF, which were experimentally obtained using cross-linking immunoprecipitation (CLIP) [14,46]. In both cases we applied the COS(WR) to the set of intergenic sequences that were experimentally selected as putative targets of the SF and a large set of exonic sequences randomly selected from human genes. As shown in Figure S2A in Additional data file 2, in the SF2/ASF experiment we did not find a significant enrichment of the SF2/ASF motif, obtained from SELEX data [26,57], within the experimental data. Nevertheless, we found that when testing the new SF2/ASF consensus motif, UGRWGVH, suggested in [46], the COS(WR) function detected a significant enrichment of the motif in experimentally selected sequences relative to a large set of random sequences from the genome. More so, the UGRWGVH motif was significantly enriched compared to all other tested motifs. Interestingly, when using the COS(WR) function we also found weaker enrichment of other SF motifs in the experimentally selected dataset. These results are consistent with the working hypothesis in the field that splicing, and specifically AS, is carried out by many SFs that work in concert to achieve fine-tuned splicing regulation [7]. To further test whether the enrichment of the motif in the putative target sequences - relative to the background - could be detected by a simple search for the consensus pattern, we screened the data searching for the same motif using the single hit approach (the *S *score). As shown in Figure S2B in Additional data file 2, when using the motif alone we did not detect a significant enrichment of the SF2/ASF motif among the CLIP target sequences. Notably, other SF motifs (such as PTB binding sites) were significantly enriched in the CLIP selected sequences also when considering a single motif, though the significance of the enrichment was reduced.

When applying the same test on NOVA-1 target sequences compared to a random set of exonic and intronic sequences, we could clearly notice a highly significant enrichment (*P *< 10^-100^) of the motif YCAY in the targets compared to the background. In the case of the NOVA-1 motif the high enrichment of the motif could be identified with the COS(WR) function but also when considering a single hit (*P *< 10^-60^). These results suggest that the YCAY motif, by itself, is sufficient to distinguish NOVA-1 targets from random sequences; this is possibly related to the high specificity of NOVA-1 to its tissue (brain) specific targets [14]. Overall, testing the COS(WR) function on CLIP data strengthens the power of the method to highlight the true SFBSs within a large set of genomic data. Nevertheless, as the CLIP data do not provide the exact location of the binding sites they could not be used to directly validate the prediction of individual SFBSs.

### Finding SFBS enrichment in alternatively spliced sequences using the COS(WR) function

**Figure 5:**
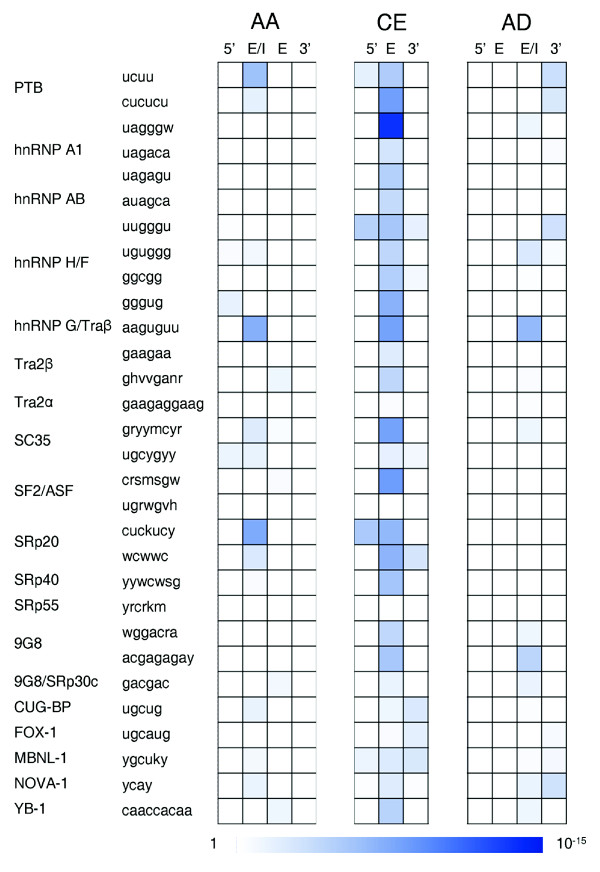
Enrichment of SFBSs in alternative exons. A heat map representing the -log_10_(*P*-value) of a series of Wilcoxon tests, comparing the normalized density of SFBS predictions in cassette exons (CE), alternative acceptors (AA), and alternative donors (AD) to a background of constitutive exons. The tests were carried out for the full exonic sequences (E), for 100-nucleotide intronic sequences (5' and 3') flanking the alternative exon and for extended regions 'exons and/or introns' (E/I). The *P*-values were corrected with the Westfall-Young procedure.

In recent years several studies have demonstrated the abundance of highly conserved sequences in the immediate regions flanking alternatively spliced exons [17,19-21,55,58]. In these studies it was suggested that both the upstream and downstream intronic regions may play a role in regulating CEs [14,16,17,19,20]. Nevertheless, in other AS modes, such as AAs and ADs, it is anticipated that only one of the introns, explicitly the one containing the AS sites, displays regulatory characteristics [21,58,59]. We therefore compared the frequency of our predicted SFBSs in CEs relative to constitutive exons and their flanking intronic sequences (as described in Materials and methods). As shown in Figure 5 (details in Table S4 in Additional data file 1), most SFBS motifs were enriched in the CEs and - to a lesser extent - in the flanking intronic sequences. Interestingly, among the SFBSs for which significant enrichment was observed in the intronic sequences, some motifs were enriched in the 5' introns (for example, UUGGGU of hnRNPH/F) and some in the 3' introns (for example, UGCAUG of FOX-1). Similar observations were recently reported in a motif search that was applied to intronic regions flanking tissue-specific CEs derived from an expression compendium of human AS events [60]. As expected, the AA exons were mainly enriched in SFBSs in the 5' introns, but not in the 3' introns. Correspondingly, the AD exons were enriched with SFBSs in the 3' introns but not in the 5' introns. As demonstrated in Figure 5, for both AAs and ADs the enrichment was specifically found in the extended region 'exon and/or intron' (E/I), which - depending on the alternative event - could be either an exonic or an intronic region. Overall, the genomic regions flanking AA and AD splicing events were less enriched with SFBSs compared to equivalent regions near constitutive events. It is important to note that when applying a similar enrichment analysis using the simple *S *function (as opposed to COS(WR)) no significant enrichment of binding sites in the AS events relative to constitutive splicing was detected (see Table S5 in Additional data file 1 and Figure S3 in Additional data file 2).

The patterns of enrichment that we observe when mapping SFBSs with the COS(WR) function on alternative exons reinforces the strength of our method in filtering true SFBSs. In addition, further interesting observations can be derived from this study. First, we observe that CEs display a larger variety of enriched SFBSs, compared to AAs and ADs, especially on the exonic sequence itself. Second, in the CE group, in several cases (such as hnRNPH/F and SRp20) binding sites of the same factor (usually different motifs) were enriched on both flanking introns. This is in accordance with AS models suggesting cross-talk between the 5' and 3' splice sites [10,61]. The enrichment of PTB binding sites in alternative versus constitutive splicing reinforces the prominent role of PTB in AS in addition to its basal role in splicing regulation of constitutive events [62]. Finally, we observed that several SFBSs were specifically enriched in the AA group (for example, SRp20) or in the AD group (for example, 9G8), while others (for example, hnRNPG/Tra2β) seem to be equally enriched in both groups (Figure 5).

### Inter-regulation among splicing factors

**Figure 6:**
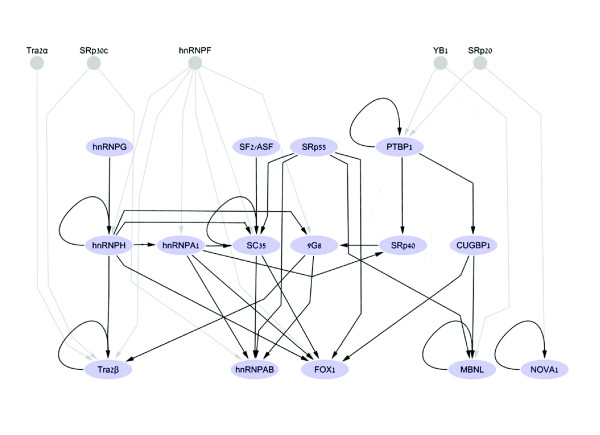
An induced subgraph of SF inter-regulation. The network represents AS regulation among SFs as predicted with the COS(WR) function. Arrows indicate that at least one of the alternative exons (and/or flanking introns) was predicted to be regulated by another factor. Light blue nodes stand for SFs that undergo AS and are thus part of the core network. SFs without AS support (the small gray nodes) are part of the extended network. The network is drawn in three layers: the upper layer displays SFs that have only out-edges (sources), the middle layer shows SFs that have both out-edges and in-edges (mixed), and the bottom layer includes SFs that have only in-edges (sinks). Graphs were drawn using Cytoscape [80].

SFs' coding transcripts have been consistently observed to be regulated by AS. In many cases negative and positive feedback via autoregulation have been observed [34,53,54,63,64]. Recent studies demonstrated that AS-related nonsense-mediated decay in SR proteins involves inter-regulatory and autoregulatory loops [65,66]. The concept of SF regulation was further strengthened by a recent computational genomic survey that demonstrated enrichment of specific SFBSs in their own coding genes [67]. In order to analyze the cross-talk (at the AS level) between the SFs within our set, we represented the relationships between the factors as a directed graph (network; Figure 6). The nodes in the graph (light blue ovals) are the SFs (both the proteins and the pre-mRNAs encoding for the SFs) and the directed edges (black arrows) denote putative regulations, predicted by the existence of a SFBS as defined by the COS(WR) function. Though the majority of SFs in our list are involved in constitutive splicing as well as in AS, to account for regulation involved in differential expression of the splicing factors, we included in the network only putative interactions with alternative spliced exons of the SF genes. To account for interactions between SFs in our list that may be involved in AS regulation but are not documented to undergo AS by themselves, we extended the core graph by adding five nodes (small grey circles) for which we could only predict out-edges (gray arrows), denoting putative interactions with other SFs via AS regulation.

Further, to study the unique properties of the SF network (including only the core network of 15 nodes for which a directed graph was constructed), we compared the network topology of the core graph to 1,000 randomly generated graphs preserving the number of nodes and edges using the Erdös-Rényi model [68]. As apparent from Table 1, the SF network demonstrated a significantly lower average path length than calculated for random graphs; however, it was not found to be highly clustered relative to random networks. Overall, the SF graph shown in Figure 6 displays a three-tier structure that is reminiscent of other regulatory networks [69]. In such a network, each node is assigned a level number: 1, 2, or 3. Generally, ignoring self loops, the three types of nodes have the following properties: level 1 nodes are 'sources', that is, nodes that have only out-going edges - these are SFs that were shown to be only regulators but are not regulated by other SFs in the core network; level 2 are 'mixed nodes', which have both in-edges and out-edges; and level 3 nodes are 'sinks', that is, nodes that have only in-going edges - these are SFs that are only regulated by other SFs and do not regulate other SFs within the network. Additionally, the network displayed many previously reported regulatory patterns such as self-splicing regulation by PTB1 [53], NOVA-1 [63] and SC35 [64]. Notably, in our network we defined an edge between SFs only for AS events in which the predicted SFBSs are enriched relative to constitutive splicing; thus, we anticipate that several autoregulatory interactions will not be reflected by the network. Obviously, our methodology will not identify autoregulation of SFs, which could occur at other levels of the gene expression pathway, such as export and translation levels (as, for example, described in [70]).

A deeper perusal of the members of the nodes in the different levels in our splicing network revealed that the sources in the network tend to be more broadly expressed SFs, such as the splicing factor SF2/ASF [71], while the sinks of the network correspond to tissue-specific splicing factors, such as the muscle- and brain-specific factor FOX-1. A specifically interesting node in the graph is PTB. As described above, PTB is well known as a basal factor, binding to polypyrimidine tracts upstream of the 3' splice sites, but it has also been shown to play a critical role in regulating tissue-specific (mainly brain) exons, including its own mRNA [53]. In the core network, PTB is found in the first layer, but it has in-edges coming from other factors (YB1, SRp20) that have not been documented as alternatively spliced. In addition, consistent with the experimental data [53], we predict that PTB is self-regulated.

**Figure 7:**
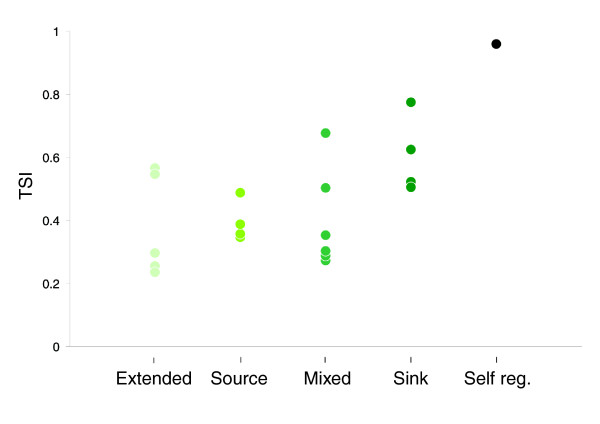
Tissue specificity of the SFs. The TSI of SFs grouped according to their positions in the network: 'extended', 'source', 'mixed', 'sink', and 'self-regulatory'. As shown, low tissue specificity is observed for the top layers while higher tissue specificity is characteristic of the bottom layers.

To further examine the relationship between the position of a factor in the graph and tissue specificity, we calculated the tissue specificity index (TSI) for the splicing factors in the network, adapted from Yanai *et al*. [72]. As illustrated in Figure 7 (for more details see Table S6 in Additional data file 1), SFs that are sinks tend to have a higher TSI compared to the sources, which generally demonstrate a low TSI. These observations coincide with the conjecture that specific factors affect a small number of targets, which are found generally in tissue-specific alternative exons; however, broadly expressed factors can regulate a wider array of targets, including alternative and constitutive exons. Additionally, these results can be explained by the fact that the more specific SFs require bulky regulatory machinery in order to maintain their specificity; therefore, they are expected to be regulated by many other factors. Interestingly, the lowest TSIs were calculated for the extended nodes, which were not included in the core network as they are not alternatively spliced. As shown in Figure 7, the brain-specific NOVA-1 splicing factor presented the highest calculated TSI. In our graph NOVA-1 displayed a single predicted self-regulatory loop, which was previously observed in an experimental assay [63], as well as an in-edge coming from SRp20 (not included in the core network). In the latter case, tissue specificity of NOVA-1 can also be explained by other levels of regulation, such as tight transcription regulation.

Finally, we wanted to examine whether specific splicing regulation events are prevalent among SF interactions. Towards this end we studied the properties of the edges of the graph. We observed that post-transcriptional regulation amongst SFs is accomplished by diverse splicing events, including CEs, ADs and AAs, and intron retention (Table S7 in Additional data file 1). We further analyzed the predicted effect of the splicing events on protein structure/function. Here again we noticed that the AS events observed in our network are predicted to have diverse outcomes, including disruptions of the RNA-binding motif, changes in the distance between adjacent RNA-binding motifs, and changes at the UTR level as in the case of several nonsense-mediated decay candidates. It is important to note that in this study we did not attempt to infer the mode of splicing regulation (that is, activation versus repression) in the SF-SF interactions, since these are dependent on the position of the SFBSs relative to the splice sites [14,19] and currently are not predictable for the vast majority of SFBSs.

## Conclusions

In this study we introduce a novel computational approach to map *cis*-regulatory elements of SFs for which a binding pattern has been previously defined from experimental data. Our newly proposed scoring function, COS(WR), which takes into account the genomic environment of a binding site, was demonstrated to achieve high specificity and sensitivity when analyzing experimentally verified SFBSs. The COS(WR) function, which considers the contribution from additional sites to the overall scoring of the binding site in a weighted manner, leverages the tendency of SFs to bind cooperatively. Furthermore, evolutionary conservation of an SFBS, which is characteristic of SFBSs in particular and regulatory motifs in general, is considered. Overall, the approach presented here is considerably different from SFBS predictors in the following aspects: in addition to SFBS similarity, it accounts for other information from the genomic environment; the COS(WR) derived scores are standardized - thus, the different SFBS prediction values are comparable between different queries and, therefore, when running the program with several SFs results can be sorted in a relative manner. The latter property makes it possible to give more probable estimations for the factors acting in the regulation of either a single AS event or a group of events (for example, alternative 3' splice sites).

By applying the COS(WR) function to map SFBSs, we were able to construct a network representing AS regulation amongst a subset of SFs. Though the details of the predicted interactions presented in the network are expected to change as more data become available, we believe that the major conclusions from this network are general and will be valid for a larger set of SFs. Interestingly, the distribution of the SFs in our network was in remarkable correlation with the tissue specificity of the factors: generally, the SFs in the top layer (the sources) showed low specificity while SFs in the bottom layer (sinks) were highly specific factors. This unique arrangement of the splicing factors suggests the existence of coordination among the different elements of the splicing regulatory machinery, not only by protein-protein interactions in the spliceosome but also via protein-RNA interactions at the post-transcription/translation levels.

## Materials and methods

### Data assembly

A total of 76 experimentally verified *cis*-regulatory sequences from human and mouse related to 20 different SFs were extracted from the AEdb regulatory motifs database [73], derived from either *in vivo *experiments or *in vitro *selective methods (Table S1 in Additional data file 1, and Additional data file 3). From this pool 30 well defined query motifs, of lengths ranging from 4 to 10 nucleotides (Table S1 in Additional data file 1), were selected. The remaining 46 sequences were used for training the algorithm (Additional data file 3). However, as some of the sequences have been shown to bind more than one SF, the final training set of 'known binding sites' included 56 samples (Additional data file 3). All sequences in the final set were extended both upstream and downstream to cover 100 bp overall; thus, each positive training sample was composed of two elements: a core 'known binding site' and the additional 'flanking sequences'.

The control set for the training processes was composed of sequences of 100 bp each, derived from the internal regions of long exons (length ≥ 1,000 nucleotides) and introns (length ≥ 10,000) (Additional data file 3). These regions were chosen as controls since they are expected to be devoid of regulatory regions [19]. Overall, the control set was composed of 353 exonic regions and 149 intronic regions (502 total). While the number of exonic regions was bounded by the length restriction, the relatively small number of intronic sequences was due to the limited availability of high-quality human/mouse alignments from internal intronic regions, which would be required for further evolutionary conservation estimates.

A background model was built to evaluate statistical significance. The background set comprised 5,000 constitutive and 1,637 alternative exons with their intronic flanking regions of length 100 bp (Table S8 in Additional data file 1), all derived from a human/mouse conserved database of alternative and constitutive exons [18].

### Defining a match to a SFBS query

To search for single SFBSs in a given sequence, the examined queries were represented as a consensus using the IUPAC definition (Table S1 in Additional data file 1). To estimate the match between the SF consensus sequence (query) and the *k*-mers in each position of the sequences (targets), a mismatch expectation (*E*_*m*_) between the query and the target was defined as:

(6) $$E_{m}=\sum_{i=1}^{n}(1-M_{i})p_{i}$$

*E*_*m *_slightly differs from an ordinary Hamming distance (namely the sum of all mismatches) as the mismatch at each position is weighted by its variability in the consensus sequence. *M*_*i *_is a Boolean variable (1 for a match and 0 for a mismatch), indicating whether the target sequence matches the query at position *i *of the *k*-mer or not. Since for most splicing factors no informative position weight matrices are currently available (except for the SR proteins for which detailed position weight matrices from SELEX data were derived [26,57]), we use a simple approach to weigh each position in the query based on the available consensus pattern. The penalty weight *p*_*i *_was defined according to the query consensus pattern given in Table S1 in Additional data file 1: it is 1 when the position in the consensus sequence is invariable and 0.25 when no restrictions are given in the consensus. The penalty weight was applied to down penalize mismatches to variable positions. Thus, for example, if the query is A[CG]A[AGC] and the 4-mer on the target sequence is AUUU, then *E*_*m *_= 0 + 0.50 + 1 + 0.33 = 1.83.

Further, a standardized score *S *was defined to evaluate the match between the query and each *k*-mer in the target sequence. Since the *E*_*m *_of a query at a certain position is highly dependent on the length and the expected nucleotide probabilities of the query, we standardized the match between the query and the *k*-mer in the target sequence as follows:

(7) $$S=\frac{E_{m}^{\max}-E_{m}}{E_{m}^{\max}}$$

- where *E*_*m *_^*max *^is the maximal mismatch expectation that can be obtained between any *k*-mer and the query. The values of *S *range from 0 to 1, increasing as the distance between the query and the *k*-mer in the target decreases. Thus, when the *k*-mer in the target sequence completely matches the query, *E*_*m *_will be 0 and *S *will equal 1. In the above example, the 4-mer AUUU will be scored (2.83 - 1.83)/2.83 = 0.353.

For defining significance, Z-scores were calculated for each query independently, relative to the background model (see the 'Data assembly' section above; Table S8 in Additional data file 1). Two different thresholds were defined: *cutoff*^*sig *^(*P*-value < 0.01) and *cutoff*^*sub *^(*P*-value < 0.025) for significant (*S*^*sig*^) and suboptimal (*S*^*sub*^) hits, respectively. Here, a mixed background model (both exons and introns taken together) was chosen since we do not observe substantial differences when considering each group separately (Table S8 in Additional data file 1).

### Testing on experimentally predicted SFBSs based on CLIP data

In order to assess the specificity and sensitivity of our method at a genome-wide scale, we employed the SF2/ASF CLIP dataset from Sanford *et al*. [46] and the NOVA-1 CLIP data from Ule *et al*. [14]. From the first set only intragenic sequences, which were identified by the CLIP technique as SF2/ASF targets, were selected (326 sequences in total) and combined with 3,260 (10-fold) random exonic sequences from the human genome. From the second set 48 validated NOVA-1 targets and 480 random exonic and intronic sequences were selected. The choice of either pure exonic or mixed (intronic/exonic) backgrounds for SF2/ASF and NOVA-1, respectively, is based on the CLIP results, where SF2/ASF targets were purely exonic while the NOVA-1 targets were mixed. The COS(WR) function was applied to predict the binding motifs from our initial SF list (Table S1 in Additional data file 1). For each independent experiment, the prediction results of SFBS scores for the experimentally chosen sequences and the random sequences were ranked. Further, the Fisher exact (hypergeometric distribution) test was applied to search which of the predicted motifs (above the COS(WR) thresholds) was significantly enriched in the CLIP derived sequences compared to random sequences.

### Enrichment analysis

To search for enrichment of SFBSs in sequences related to AS events versus constitutive splicing events, three different sets of human/mouse conserved alternative exons were tested: a set of 983 CEs; 439 alternative acceptors; and 198 alternative donors [18]. All the exon and intron (with masked splice sites) sets were compared with a non-parametric Wilcoxon test to a set of 5,000 randomly chosen constitutive exons, also conserved between human and mouse [18]. All the obtained *P*-values were corrected using the Westfall-Young procedure [74].

### Splicing networks

Interactions between splicing factors (via AS) were represented by a directed graph G = (V, E) where the SFs are the nodes in V and the edges in E reflect interactions, as follows: a directed edge from SF *s*_*1*_(the candidate regulator) to SF *s*_*2*_(the target transcript) exists if at least one alternative exon of *s*_*2*_was significantly enriched in the SFBSs of *s*_*1*_. To establish interactions, the alternative exons (and the flanking introns) of the SFs were queried with 30 SFBS motifs. Alternative exons were defined based on annotations from RefSeq [75], H-DBAS [76], and dbCASE [77]. In the latter, we considered AS events with ≥ 4 expressed sequence tags per isoform. Under these conditions, we observe a large extent of overlap between annotations in all the databases. Fisher's exact tests were performed for each independent motif to define the number of significant hits that minimizes the *P*-value (in exons and introns separately) when comparing alternative to constitutive splicing events. In other words, the threshold corresponds to the minimal number of hits that is required to establish a regulatory interaction in either exons or introns. Motifs with a *P*-value > 0.05 (that is, not enriched) were not queried in the analysis.

The properties of this graph (network) were compared to 1,000 randomly generated graphs with the same number of nodes and edges using the Erdös-Rényi model [68]. Five SFs for which alternative exons were not documented (Tra2α, SRp20, SRp30c, hnRNPF, YB1) were excluded from the network analysis since they can only have out-edges (predicted to regulate other factors via AS but not *vice versa*). The following topological properties were calculated for each graph G. First, the diameter (*D*), defined as the length of the longest shortest path between any two nodes in V. Second, the average path length (*L*), defined as the average of path lengths taken over all pairs of nodes for which a directed path exists, calculated as:

(8) $$L=\frac{1}{Np}\sum_{\begin{array}{l} u,v\in V\\ u\to v \end{array}}dist(u,v)$$

- where *N*_*p *_represents the number of connected pairs of nodes in the graph, and *dist*(*u*,*v*) is the length of the shortest path between nodes *u *and *v *if one exists. Third, the clustering coefficient (*C*), which is the average value of the individual clustering coefficients (c) of all the nodes in the graph; the latter (c) is defined for a node *v *as the fraction of the number of edges among *v*'s neighbors out of all possible pairs of such neighbors. Thus, *C *is defined as:

(9) $$C=\frac{1}{N}\sum_{v\in V}\frac{n_{v}}{N_{v}(N_{v}-1)/2}$$

- where *N *is the number of nodes (vertices) in the graph, *N*_*v *_is the number of neighbors of node *v*, and *n*_*v *_is the actual number of edges between the neighbors of node *v*. The analyses were performed with the R software environment for statistical computing release 2.5.1 [78] and the igraph contributed (0.4.3) package using the functions: erdos.reni.game, diameter, average.path.length and transitivity.

### Tissue specificity index

The TSI of the splicing factors was calculated using the GPL96-GDS596-MAS5 microarrays dataset [79]. SF expression levels for a total of 28 normal tissues were used for calculating each TSI; cancer and fetal tissues were removed. Further, the expression levels were log transformed and binned into ten groups ranging from 0 to 1 for every sample independently.

The TSI was adapted from the TSIhvr value, defined by Yanai *et al*. [72]. As in the TSIhvr, the expression profile for each SF was first normalized by dividing each intensity by the highest intensity of that profile, as follows:

(10) $$TSI_{hvr}=\frac{\sum_{i}^{N}1-x_{i}}{N-1}$$

- where N is the number of tissues (28) and x is the normalized expression vector.

## Availability

The method presented here is embodied in a software package called Splicing Factor Finder (SFF), which is available in Additional data file 4 as a standalone download suitable for running under the Linux OS.

## Abbreviations

AA: alternative acceptor; AD: alternative donor; AS: alternative splicing; CE: cassette exon; CLIP: cross-linking immunoprecipitation; COS: Conservation Of Score; hnRNP: heterogeneous nuclear ribonucleoprotein; M: median; PTB: polypyrimidine tract-binding protein; SF: splicing factor; SFBS: splicing factor binding site; SR: serine-arginine-rich; SS: sum of scores; TPR: true positive rate; TSI: tissue specificity index; UTR: untranslated region; WA: weighted average; WR: weighted rank.

## Authors' contributions

MA participated in the design and development of the computational methodology, carried out the predictions and statistical analyses, and drafted the manuscript. HDE carried out the network analysis. RYP advised on the network design and analysis. YMG conceived and coordinated the study and wrote the manuscript. All authors read the manuscript and participated in the revisions that produced its final form.

## Additional data files

The following additional data are available with the online version of this paper: a PDF including Tables S1-S8 (Additional data file 1); a PDF including Figures S1-S3 (Additional data file 2); a detailed table of all experimentally defined SFBSs used for training and testing (Additional data file 3); a compressed file of the SFF standalone download, suitable for running under the Linux OS (Additional data file 4).

## Supplementary Material

Additional data file 1 Table S1 includes a list of binding site motifs for known SFs used for training and testing the method. Table S2 summarizes the training results using different estimators and thresholds. Table S3 lists the thresholds used for the COS(WR) function for each binding site motif. Tables S4 and Table S5 display detailed results for the enrichment tests performed for AAs, CEs, and ADs, applying the COS(WR) and single score (*S*), respectively. Table S6 displays the values of the TSI calculated for the different SFs. Table S7 presents the details of the predicted SF-SF interactions. Table S8 displays the values for the background model calculated for the Single Scores (*S*). Click here for file

Additional data file 2 Figure S1 illustrates the TPR of different multiplicity estimators (WR, WA, M, SS and S) calculated at a fixed false positive rate of 0.01. TPRs were calculated with and without the COS. Figure S2 demonstrates the analysis of the SF2/ASF and NOVA-1 CLIP datasets, when applying (a, c) COS(WR) and (b, d) Single Scores. Figure S3 is a heat map representing the calculated enrichment of SFBSs around different alternative events, when applying Single Scores (*S*) only. Click here for file

Additional data file 3 Experimentally defined SFBSs used for training and testing. Click here for file

Additional data file 4 SFF standalone download, suitable for running under the Linux OS. Click here for file
