# Supplementary material for: A computational approach for genome-wide mapping of splicing factor binding sites
Source: Genome Biol. 2009 Mar 18;10(3):R30. doi: 10.1186/gb-2009-10-3-r30 (PMC2691001; doi:10.1186/gb-2009-10-3-r30)
Supplement: Additional data file 1 — Table S1 includes a list of binding site motifs for known SFs used for training and testing the method. Table S2 summarizes the training results using different estimators and thresholds. Table S3 lists the thresholds used for the COS(WR) function for each binding site motif. Tables S4 and Table S5 display detailed results for the enrichment tests performed for AAs, CEs, and ADs, applying the COS(WR) and single score (S), respectively. Table S6 displays the values of the TSI calculated for the different SFs. Table S7 presents the details of the predicted SF-SF interactions. Table S8 displays the values for the background model calculated for the Single Scores (S). [file gb-2009-10-3-r30-S1.pdf]

Table S1: List of splicing factors and splicing factor binding site motifs.

Factor	Motif	Reference
PTB	ucuu	[53]
	cucucu	[35, 37]
hnRNPA1	uagggw	[31]
	uagaca	[42]
	uagagu	[34]
hnRNPAB	auagca	[40]
hnRNPH/F	uugggu	[41]
	uguggg	[36]
	ggcgg	[45]
	gggug	[38]
hnRNPG/Tra2beta	aaguguu	[44]
Tra2beta	gaagaa	[51]
	ghvvganr	[52]
Tra2alpha	gaagaggaag	[48]
SC35	gryymcyr	[26]
	ugcygyy	[47]
SF2/ASF	crsmsgw	[26]
	ugrwgvh	[46]
SRp20	cuckucy	[32]
	wcwwc	[33]
SRp40	yywcwsg	[26]
SRp55	yrcrkm	[26]
9G8	wggacra	[32]
	acgagagay	[33]
9G8/SRp30c	gacgac	[33]
CUG-BP	ugcug	[11, 50]
FOX-1	ugcaug	[43]
MBNL-1	ygcuky	[39]
NOVA-1	ycay	[14]
YB-1	caaccacaa	[49]

Table S2 : Training results

Motifs	*Estimator*	$cutoff^{sig}$	$cutoff^{sub}$	*w*	Avg TPR	Sd TPR
SFBS	S	0.01	0.025	50	0.681	0.038
SFBS	$COS(SS_{w})$	0.01	0.025	50	0.597	0.173
SFBS	$COS(M_{w})$	0.01	0.025	50	0.809	0.063
SFBS	$COS(WA_{w})$	0.01	0.025	50	0.817	0.054
SFBS	$COS(WR_{w,a=2})$	0.01	0.025	50	0.929	0.016
SFBS	$COS(WR_{w,a=3})$	0.01	0.025	50	0.911	0.033
SFBS	SS_{w}	0.01	0.025	50	0.644	0.028
SFBS	M_{w}	0.01	0.025	50	0.755	0.061
SFBS	WA_{w}	0.01	0.025	50	0.788	0.049
SFBS	$WR_{w,a=2}$	0.01	0.025	50	0.894	0.037
SFBS	$WR_{w,a=3}$	0.01	0.025	50	0.892	0.041
SFBS	$COS(WR_{w,a=2})$	0.01	0.01	50	0.841	0.026
SFBS	$COS(WR_{w,a=2})$	0.01	0.05	50	0.870	0.052
SFBS	$COS(WR_{w,a=2})$	0.025	0.025	50	0.504	0.025
SFBS	$COS(WR_{w,a=2})$	0.01	0.025	10	0.838	0.031
SFBS	$COS(WR_{w,a=2})$	0.01	0.025	20	0.878	0.023
SFBS	$COS(WR_{w,a=2})$	0.01	0.025	40	0.890	0.023
SFBS	$COS(WR_{w,a=2})$	0.01	0.025	50	0.916	0.021
SFBS	$COS(WR_{w,a=2})$	0.01	0.025	60	0.919	0.028
SFBS	$COS(WR_{w,a=2})$	0.01	0.025	70	0.898	0.045
SFBS	$COS(WR_{w,a=2})$	0.01	0.025	80	0.894	0.055
SFBS	$COS(WR_{w,a=2})$	0.01	0.025	90	0.873	0.061
SFBS	$COS(WR_{w,a=2})$	0.01	0.025	100	0.860	0.055
Promoter	$COS(WR_{w,a=2})$	0.01	0.025	50	0.593	0.025
UTR	$COS(WR_{w,a=2})$	0.01	0.025	50	0.550	0.012
Promoter	$WR_{w,a=2}$	0.01	0.025	50	0.555	0.030
UTR	$WR_{w,a=3}$	0.01	0.025	50	0.566	0.043

Summary of training results. The average percent of true positives (%TP) (100 iterations) at a fixed false positive rate of 1% when training the data with different multiplicity estimators: Weighted Rank (WR), Weighted Average (WA), Median (M), Sum of Score (SS), with and without the COS function in comparison to the Single Score (S). In addition, tests were conducted with variable parameters: window size (w), significant cutoff (cutoffsig), suboptimal cutoff (cutoffsub) and a values (only for WR). As a control, we trained the data with UTR and promoter motifs instead of SFBS.

Table S3:Thresholds used for COS(WR)

factor	Query	Cutoff Exon	Cutoff Intron	Cutoff ext. Exon	Cutoff Mixed
PTB	ucuu	0.749	0.781	0.828	0.747
	cucucu	0.666	0.689	0.729	0.667
hnRNP A1	uagggw	0.583	0.5	0.584	0.583
	uagaca	0.583	0.5	0.584	0.544
	uagagu	0.602	0.616	0.642	0.602
hnRNP AB	auagca	0.583	0.5	0.5835	0.544
hnRNP H/F	uugggu	0.5	0.465	0.6	0.5
	uguggg	0.514	0.514	0.6	0.514
	ggcgg	0.583	0.545	0.584	0.583
	gggug	0.5	0.5	0.583	0.5
hnRNP G/Tra2β	aaguguu	0.357	0.357	0.357	0.357
Tra2β	gaagaa	0.729	0.584	0.749	0.667
	ghvvganr	0.775	0.675	0.825	0.737
Tra2α	gaagaggaag	0.612	0.5	0.6485	0.575
SC35	gryymcyr	0.6	0.45	0.65	0.594
	ugcygyy	0.616	0.545	0.659	0.591
SF2/ASF	crsmsgw	0.681	0.583	0.727	0.639
	ugrwgvh	0.748	0.607	0.761	0.688
SRp20	cuckucy	0.625	0.66	0.6895	0.625
	wcwwc	0.714	0.643	0.75	0.643
SRp40	yywcwsg	0.681	0.6	0.722	0.639
SRp55	yrcrkm	0.748	0.621	0.714	0.68
9G8	wggacra	0.485	0.396	0.544	0.468
	acgagagay	0.625	0.5	0.656	0.583
9G8/SRp30c	gacgac	0.5	0.334	0.5105	0.5
CUG-BP	ugcug	0.6	0.411	0.6	0.5
FOX-1	ugcaug	0.593	0.544	0.625	0.583
MBNL-1	ygcuky	0.668	0.639	0.736	0.639
NOVA-1	ycay	0.812	0.708	0.854	0.75
YB-1	caaccacaa	0.573	0.473	0.594	0.539

Table S4: Enrichment of SFBSs in alternative exons

SF	SFBS	Alternative Acceptor				Cassette Exon			Alternative Donor			
		5' Intron	Ex/Int	Exon	3' Intron	5' Intron	Exon	3' Intron	5' Intron	Exon	Ex/Int	3' Intron
PTB	ucuu	-2.341	5.823	-2.215	-1.033	1.761	4.872	-2.42	-2.459	-2.585	-0.727	3.34
	cucucu	-2.294	1.743	-2.33	-2.181	-0.775	8.106	-1.221	-2.131	-2.501	-1.771	2.474
hnRNP A1	uagggw	-1.127	-1.689	-1.184	-2.074	-2.218	13.279	-2.329	-1.725	-0.771	1.131	-0.377
	uagaca	-2.742	-2.549	-1.074	-2.336	-2.774	2.91	-2.93	-2.949	-2.6	-1.975	0.527
	uagagu	-0.343	-1.858	-1.393	-1.495	-0.044	4.64	-2.195	-2.151	-2.232	-0.89	-0.538
hnRNP AB	auagca	-3.166	-2.762	-2.933	-2.868	-2.58	3.66	-2.85	-2.958	-1.63	-2.158	-1.503
hnRNP H/F	uugggu	0.282	-0.763	-2.637	-2.301	4.466	5.318	1.669	-1.076	-2.516	-1.606	3.162
	uguggg	0.515	0.971	-1.578	-2.26	-1.246	3.97	-0.548	-0.857	-2.282	2.427	0.554
	ggcgg	-0.465	-0.4	-1.609	-2.064	-1.479	4.657	0.857	-1.146	-0.509	0.257	-0.163
	gggug	1.538	-1.642	-2.244	-1.995	-1.916	6.802	-2.018	-2.046	-2.439	-0.149	-1.331
hnRNP G/Traβ	aaguguu	-0.971	6.934	-2.575	-2.832	-1.124	7.865	-1.742	-2.687	-1.345	6.268	-0.072
Tra2β	gaagaa	-1.599	-2.173	-0.825	-2.247	-2.274	2.155	-1.614	-1.477	-1.705	0.013	-1.139
	ghvvganr	-1.522	-3.015	1.151	-2.387	-3.236	3.954	-3.226	-2.986	-2.181	0.448	-3.029
Tra2α	gaagaggaag	-1.992	-2.432	-0.489	-1.425	-2.412	0.468	-2.219	-2.315	-1.186	0.311	-1.685
SC35	gryymcyr	-0.368	2.242	0.385	-2.127	-2.169	7.833	-1.521	-1.541	-0.876	1.133	-1.242
	ugcygyy	1.3	1.518	-0.694	-1.97	-0.381	1.69	0.95	-0.719	-2.066	-0.518	-1.322
SF2/ASF	crsmsgw	-1.067	-3.197	0.484	-3.32	-3.51	8.199	-3.383	-2.5	-1.738	-2.017	-2.666
	ugrwgvh	-2.167	-2.769	-3.286	-3.687	-3.944	-0.768	-2.033	-3.299	-3.275	-2.706	-1.01
SRp20	cuckucy	-2.588	7.334	-0.117	-2.326	5.011	6.388	-1.267	-1.97	-2.552	-1.791	-0.612
	wcwwc	-2.799	2.445	-0.76	-1.757	-0.162	6.584	2.802	-2.762	-1.11	-0.569	-0.541
SRp40	yywcwsg	-1.717	0.478	-2.259	-1.986	-2.516	5.52	-2.023	-2.608	-2.388	-0.187	-0.157
SRp55	yrcrkm	-2.374	-0.955	-1.096	-2.733	-2.293	0.097	-2.006	-1.388	-1.514	-2.386	-1.815
9G8	wggacra	-2.312	-2.514	-2.599	-2.505	-2.506	3.904	-2.426	-2.297	-1.186	1.135	-1.576
	acgagagay	-1.911	-2.076	-0.517	-2.081	-2.23	5.25	-0.4	-1.621	-1.396	4.178	-1.659
9G8/SRp30c	gacgac	-2.213	-1.532	0.96	-2.52	-2.999	1.444	-2.863	-2.06	-1.099	1.372	-0.401
CUG-BP	ugcug	-1.982	1.511	-2.514	-3.701	-0.435	1.128	2.449	-2.901	-3.028	0.302	-0.483
FOX-1	ugcaug	-1.86	-1.123	-1.034	-2.848	-2.063	0.496	1.779	-2.77	-1.66	-2.091	0.602
MBNL-1	ygcuky	-0.289	0.904	-0.681	-2.242	1.315	2.349	2.512	-1.967	-1.754	0.45	0.78
NOVA-1	ycay	-2.28	1.422	-1.359	-2.496	0.252	2.143	0.328	-1.946	-1.56	1.434	3.029
YB-1	caaccacaa	-1.501	-0.075	1.183	-1.689	-1.491	4.336	-1.712	-1.806	-1.048	1.192	-1.629

Enrichment of SFBSs in alternative exons. The table displays details of the Wilcoxon tests, comparing the normalized density of SFBSs in Cassette Exons (CE), Alternative Acceptors (AA) and Alternative Donors (AD) to a background of Constitutive Exons. The tests were carried out for the exonic and intronic sequences separately. p-values were corrected with the Westfall-Young procedure. The numbers correspond to the Westfall-Young corrected -log(p-value) of the Wilcoxon test.

Table S5: Enrichment of SFBSs in alternative exons applying Single Scores (S)

		Alternative Acceptor				Cassette Exon			Alternative Donor			
SF	SFBS	5' Intron	Ex/Int	Exon	3' Intron	5' Intron	Exon	3' Intron	5' Intron	Exon	Ex/Int	3' Intron
PTB	ucuu	-2.531	1.533	-1.917	-2.477	2.187	6.974	-2.261	-2.608	-2.03	0.775	-2.148
	cucucu	0.888	-2.362	-2.091	-2.191	-0.333	-1.067	-2.303	-2.315	-2.664	-2.681	-2.647
	uagggw	-2.32	-1.719	-2.356	-2.119	-2.355	-2.313	-2.27	-2.263	-2.325	-2.379	-2.283
hnRNP A1	uagaca	-2.979	-2.298	-3.031	-3.003	-3.011	-2.389	-2.616	-2.921	-2.961	-3.147	-2.945
	uagagu	-2.241	-2.396	-2.361	-2.27	-2.293	-2.177	-2.306	-2.414	-2.356	-1.094	-2.191
hnRNP AB	auagca	-3.051	-1.927	-3.203	-3.08	-3.117	-1.832	-2.699	-3.03	-2.918	-2.895	-3.037
	uugggu	-2.665	-2.695	-2.221	-2.617	-2.644	-1.519	-2.453	-2.608	-2.682	-2.729	-1.56
hnRNP H/F	uguggg	-1.848	-1.137	-2.094	-2.423	-1.907	-1.44	-2.139	-2.338	-2.433	-0.63	-1.861
	ggcgg	-1.902	-1.767	-1.907	-2.487	-1.319	0.13	-2.215	-1.727	0.622	-1.014	-1.554
	gggug	-0.8	0.156	-2.249	-1.694	-2.172	0.54	-2.389	-2.571	-2.675	-0.59	-1.543
hnRNP G/Traβ	aaguguu	-2.685	-2.341	-2.015	-2.688	-2.699	-2.716	-2.157	-2.652	-2.875	-0.858	0.548
Tra2β	gaagaa	-2.291	-2.13	0.658	-1.657	-2.33	0.14	-2.188	-2.252	-1.687	-1.572	-2.055
	ghvvganr	-3.171	-2.227	-1.901	-3.149	-3.226	3.8	-3.051	-2.648	-2.662	0.721	-3.216
Tra2α	gaagaggaag	-2.579	-2.579	-2.248	-2.579	-2.579	-2.035	-2.579	-2.579	-2.579	-2.579	-2.579
SC35	gryymcyr	-2.044	-1.698	-1.73	-1.016	-2.215	-1.551	-1.778	-1.463	-1.985	-1.468	-1.685
	ugcygyy	-1.84	-0.378	-0.743	-1.778	-1.707	-0.261	-0.839	-2.542	-2.327	-2.603	-2.287
SF2/ASF	crsmsgw	-3.492	-3.908	-2.285	-3.916	-3.431	-0.876	-2.645	-3.456	-2.353	-1.973	-3.193
	ugrwgvh	-2.944	-3.239	-3.119	-3.571	-3.972	0.729	-3.346	-3.193	-3.527	-2.49	-3.923
SRp20	cuckucy	-3.105	-0.439	-3.057	-2.886	-2.259	-1.782	-2.457	-2.843	-3.115	-2.86	-3.029
	wcwwc	-3.086	1.204	-2.324	-2.493	-1.715	0.57	-2.177	-2.949	-2.28	0.242	-1.669
SRp40	yywcwsg	-2.802	-1.453	-1.869	-2.631	-2.815	3.118	-2.84	-2.826	-1.774	-0.851	-0.51
SRp55	yrcrkm	-3.013	-0.421	2.532	-2.966	-2.99	2.481	-2.686	-2.638	-2.609	-1.536	-2.71
9G8	wggacra	-2.599	-2.523	-2.2	-2.415	-2.599	-2.344	-1.714	-2.599	-2.284	-0.74	-2.394
	acgagagay	-2.25	-2.25	-2.25	-2.25	-2.25	-2.25	-2.25	-2.25	-2.25	-2.25	-2.25
9G8/SRp30c	gacgac	-3.033	-3.033	-2.869	-3.033	-2.887	-2.026	-3.033	-2.88	-2.843	-3.033	-3.033
CUG-BP	ugcug	-3.209	-0.701	-1.684	-1.888	-3.127	-0.822	-1.097	-3.673	-3.379	-3.138	-3.47
FOX-1	ugcaug	-2.9	-2.979	-2.85	-2.925	-1.838	-2.525	-1.457	-2.873	-2.874	-3.001	-2.921
MBNL-1	ygcuky	-0.822	1.604	0.876	-1.731	1.821	2.21	-0.135	-1.652	-1.483	-2.147	-1.617
NOVA-1	ycay	-2.555	5.164	-1.679	-2.453	-0.798	1.099	-1.584	-2.283	-2.246	2.691	-1.585
YB-1	caaccacaa	-2.153	-2.153	-2.153	-2.153	-2.153	-1.989	-2.153	-2.153	-2.153	-2.153	-2.153

Enrichment of SFBSs in alternative exons when applying Single Scores (S). The table displays details of the Wilcoxon tests, comparing the normalized density of SFBSs in Cassette Exons (CE), Alternative Acceptors (AA), and Alternative Donors (AD) to a background of Constitutive Exons. The tests were carried out for the exonic and intronic sequences separately. p-values were corrected with the Westfall-Young procedure. The numbers correspond to the Westfall-Young corrected -log(p-value) of the Wilcoxon test.

Table S6: Tissue specificity index (TSI) of the splicing factors

Splicing Factor	type	TSI
9G8	Mixed	0.676
CUGBP1	Mixed	0.272
FOX1	Sink	0.624
hnRNPG	Source	0.388
HNRPA1	Mixed	0.302
HNRPAB	Sink	0.505
HNRPF	Extended	0.566
HNRPH3	Mixed	0.502
MBNL1	Sink	0.775
NOVA1	Self Regulation	0.958
PTBP1*	Source	0.356
SC35	Mixed	0.288
SF2/ASF	Source	0.486
SRp20	Extended	0.296
SRp30c	Extended	0.254
SRp40	Mixed	0.353
SRp55	Source	0.345
Tra2β	Sink	0.522
Tra2α	Extended	0.546
YB1	Extended	0.235

*Only in the core network

Table S7: Details of predicted SF to SF interactions from the network

Factor	Target	Hit Position	Event Type	Coordinates	5' UTR	ORF	3' UTR	Description
hnRNPH/F	9G8	5' Exon	CE	chr2:38826789-38826825:-	0	1	0	SR motif affected
SRp40	9G8	E	IR	chr2:38829224-38829299:-	0	1	0	SR motif affected
hnRNPH/F	FOX1	5' Exon	CE	chr22:34482097-34482137:-	0	1	0	
hnRNPA1	FOX1	E	AA	chr22:34507591-34507739:-	0	1	0	Single amino acid variation
CUG-BP,SC35,SRp55	FOX1	E	IR	chr22:34472465-34472554:-	0	1	0	NMD candidate
hnRNPH/F	FOX1	5' Exon	IR	chr22:34472465-34472554:-	0	1	0	NMD candidate
hnRNPH/F	hnRNPA1	E	CE	chr12:52963129-52963285:+	0	1	0	G-rich sequence affected
9G8,SRp30c,hnRNPA1,SRp55	hnRNPAB	E	CE	chr5:177569738-177569879:+	0	1	0	G-rich sequence affected
SC35	hnRNPAB	5' Exon	CE	chr5:177569738-177569879:+	0	1	0	G-rich sequence affected
hnRNPG	hnRNPH3	3' Exon	IR	chr10:69766961-69767096:+	1	1	0	RRM motif affected
hnRNPH/F	hnRNPH3	5' Exon	IR	chr10:69767620-69767759:+	1	1	0	RRM motif affected
hnRNPH/F	hnRNPH3	3' Exon	IR	chr10:69767620-69767759:+	1	1	0	RRM motif affected
SRp55	MBNL1	E	CE	chr3:153633195-153633399:+	0	1	0	
MBNL	MBNL1	5' Exon	CE,ME	chr3:153535589-153535709:+	1	0	0	
CUG-BP	MBNL1	E	CE	chr3:153658608-153658672:+	0	1	0	Affects carboxyl end of the protein
YB1	MBNL1	E	CE	chr3:153656020-153656056:+	0	1	0	
MBNL	MBNL1	5' Exon	CE	chr3:153647182-153647236:+	0	1	0	
MBNL	MBNL1	5' Exon	CE	chr3:153648098-153648252:+	0	1	0	
YB1	MBNL1	E	CE	chr3:153656745-153656840:+	0	1	0	
NOVA1	NOVA1	3' Exon	CE	chr14:26011365-26011437:-	0	1	0	Affects the distance between KH domains
NOVA1,SRp20	NOVA1	E	CE	chr14:26011365-26011437:-	0	1	0	Affects the distance between KH domains
PTB,YB1	PTBP1	E	CE	chr19:758868-758902:+	0	1	0	RRM motif affected
SRp20,	PTBP1	5' Exon	CE	chr19:758868-758902:+	0	1	0	RRM motif affected
hnRNPA1,hnRNPH/F,SC35,SF2ASF,SRp55	SC35	E	CE,AD,IR	chr17:72244475-72244503:-	0	1	0	G-rich and SR affected
SC35	SC35	5' Exon	CE,AD,IR	chr17:72244475-72244503:-	0	1	0	G-rich and SR affected
hnRNPA1	SRp40	E	IR	chr14:69305651-69305721:+	0	1	1	Affects the distance between RRMs. NMD candidate
PTB	SRp40	5' Exon	IR	chr14:69306936-69307010:+	1	1	0	
9G8,SRp30c,hnRNPH/F,Tra2β,Tra2β	Tra2β	E	CE	chr3:187119918-187119978:-	0	1	0	G-rich sequence affected

Details of predicted SF to SF interactions from the network. The table shows details regarding the target predicted regulators, the position of the predicted hit (exonic, intronic), the type of alternative exon (CE= cassette exons, AA= alternative acceptors, AD= alternative donors, MS= mutual exclusive exon, IR= intron retention), the genomic positions of the target (hg18), the portion of the transcript affected by alternative splicing (UTRs and ORFs), and a short description of the effect of the alternative splicing event of the target.

Table S8: Background model calculated for single score S

factor	query	Exon		Intron		Mixed	
PTB	ucuu	0.235	±0.221	0.256	±0.2275	0.254	±0.228
	cucucu	0.242	±0.211	0.246	±0.206	0.245	±0.205
hnRNP A1	uagggw	0.276	±0.174	0.276	±0.1765	0.276	±0.177
	uagaca	0.26	±0.177	0.2585	±0.179	0.259	±0.179
	uagagu	0.254	±0.174	0.2585	±0.1765	0.258	±0.176
hnRNP AB	auagca	0.26	±0.179	0.2585	±0.1775	0.259	±0.178
hnRNP H/F	uugggu	0.243	±0.171	0.252	±0.1755	0.25	±0.176
	uguggg	0.246	±0.179	0.247	±0.181	0.246	±0.181
	ggcgg	0.248	±0.191	0.2335	±0.1895	0.234	±0.191
	gggug	0.248	±0.193	0.2425	±0.195	0.242	±0.195
hnRNP G/Tra2β	aaguguu	0.251	±0.166	0.2595	±0.1675	0.259	±0.167
Tra2β	gaagaa	0.27	±0.197	0.262	±0.1925	0.263	±0.193
	ghvvganr	0.462	±0.167	0.4495	±0.1665	0.45	±0.167
Tra2α	gaagaggaag	0.265	±0.153	0.2555	±0.1505	0.256	±0.15
SC35	gryymcyr	0.396	±0.174	0.3905	±0.171	0.391	±0.171
	ugcygyy	0.305	±0.189	0.3085	±0.1835	0.308	±0.184
SF2/ASF	crsmsgw	0.388	±0.179	0.375	±0.177	0.376	±0.177
	ugrwgvh	0.378	±0.2	0.3785	±0.194	0.378	±0.193
SRp20	cuckucy	0.276	±0.173	0.283	±0.175	0.282	±0.176
	wcwwc	0.355	±0.214	0.359	±0.209	0.359	±0.209
SRp40	yywcwsg	0.381	±0.183	0.382	±0.181	0.382	±0.182
SRp55	yrcrkm	0.428	±0.193	0.422	±0.1905	0.422	±0.191
9G8	wggacra	0.303	±0.179	0.295	±0.1745	0.296	±0.175
	acgagagay	0.275	±0.146	0.2675	±0.1455	0.268	±0.146
9G8/SRp30c	gacgac	0.255	±0.176	0.2445	±0.17	0.245	±0.17
CUG-BP	ugcug	0.235	±0.179	0.2445	±0.183	0.244	±0.184
FOX-1	ugcaug	0.248	±0.187	0.2505	±0.1835	0.25	±0.183
MBNL-1	ygcuky	0.318	±0.202	0.3255	±0.198	0.325	±0.198
NOVA-1	ycay	0.328	±0.237	0.3285	±0.2355	0.329	±0.236
YB-1	caaccacaa	0.259	±0.15	0.251	±0.1485	0.253	±0.149
