# Supplementary material for: A computational approach for genome-wide mapping of splicing factor binding sites
Source: Genome Biol. 2009 Mar 18;10(3):R30. doi: 10.1186/gb-2009-10-3-r30 (PMC2691001; doi:10.1186/gb-2009-10-3-r30)
Supplement: Additional data file 2 — Figure S1 illustrates the TPR of different multiplicity estimators (WR, WA, M, SS and S) calculated at a fixed false positive rate of 0.01. TPRs were calculated with and without the COS. Figure S2 demonstrates the analysis of the SF2/ASF and NOVA-1 CLIP datasets, when applying (a, c) COS(WR) and (b, d) Single Scores. Figure S3 is a heat map representing the calculated enrichment of SFBSs around different alternative events, when applying Single Scores (S) only. [file gb-2009-10-3-r30-S2.pdf]

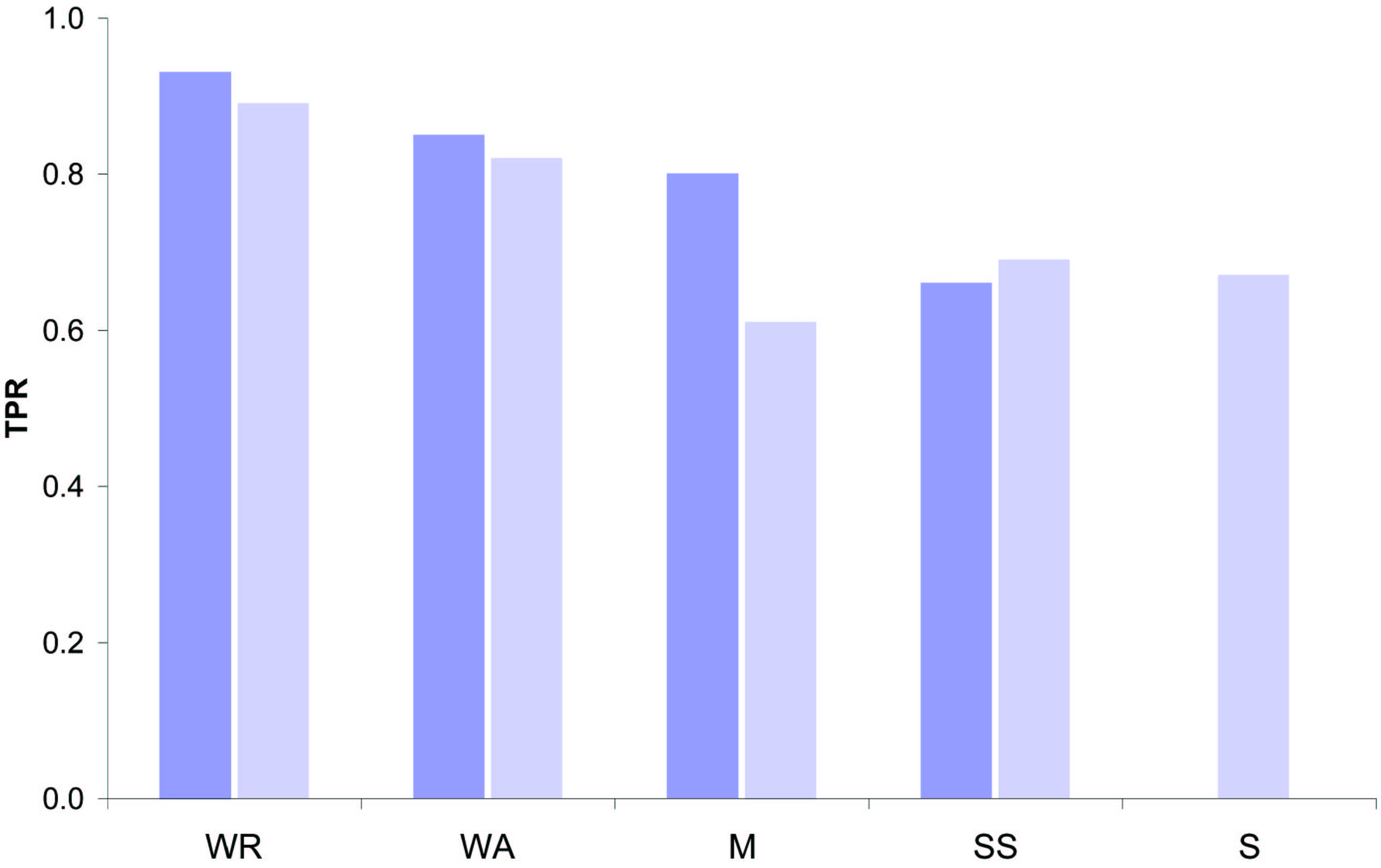

Figure S1: Sensitivity of multiplicity estimators. The true positive rate (TPR) at a fixed false positive rate of 0.01 when training the full dataset (56 positives and 502 negatives) with five different estimators: Weighted Rank (WR), Weighted Average (WA), Median (M), Sum of all the Scores (SS), and Single Scores (S). For each estimator the TPR was calculated when considering (dark columns) or not considering (light columns) the Conservation Of Score (COS).

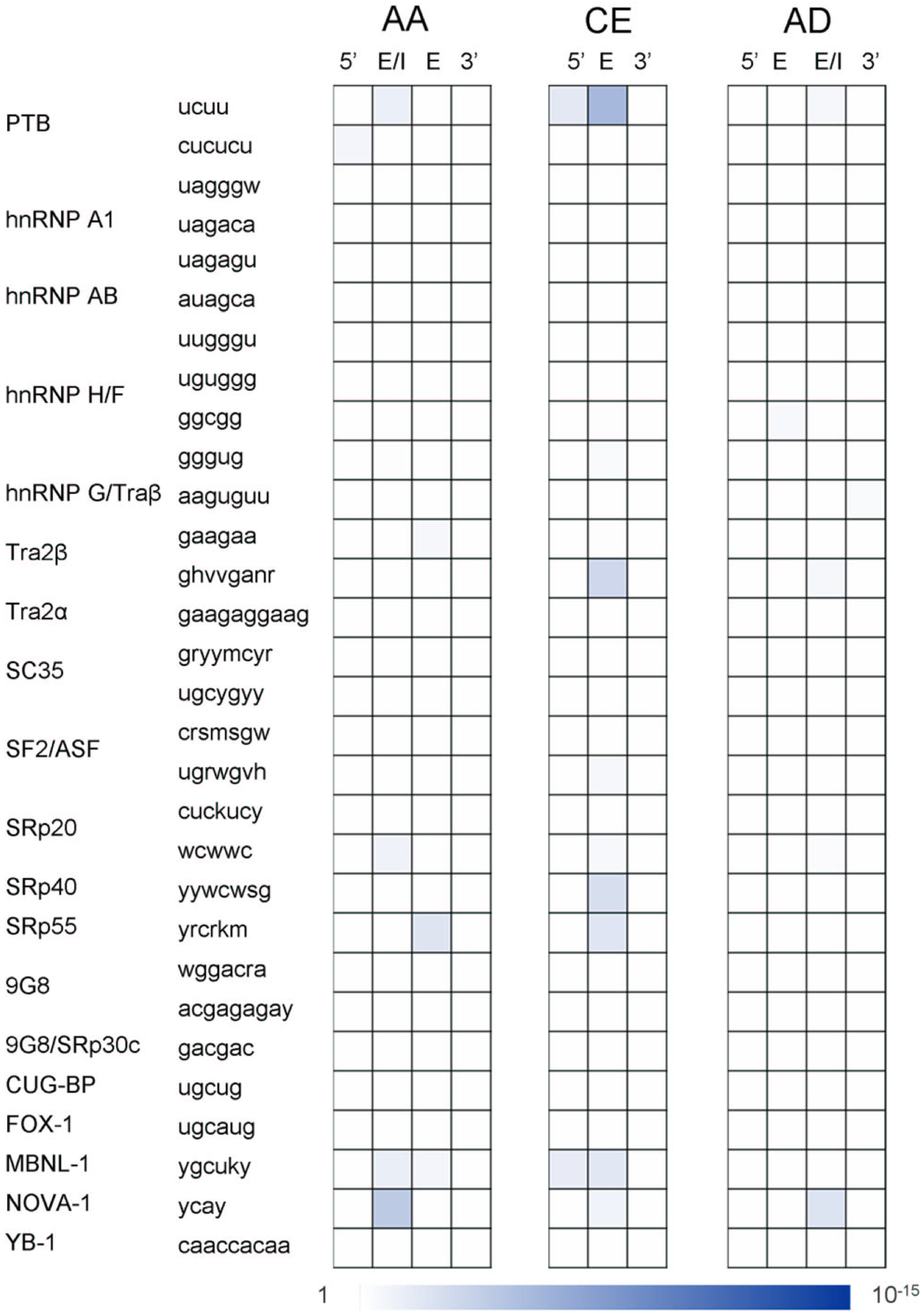

Figure S3: Enrichment of SFBS in alternative exons applying Single Scores (S). A heat map representing the *-log10(p-value)* of a series of Wilcoxon tests, comparing the normalized density of SFBS predictions in cassette exons (CE), Alternative Acceptors (AA), and Alternative Donors (AD) to a background of Constitutive Exons. For CE the tests were carried out for the full exonic sequences and for 100 nt intronic sequences (5' and 3') flanking the alternative exons. For alternative acceptors and alternative donors the alternative exon/intron region was analyzed separately. The *p*-values were corrected with the Westfall-Young procedure.

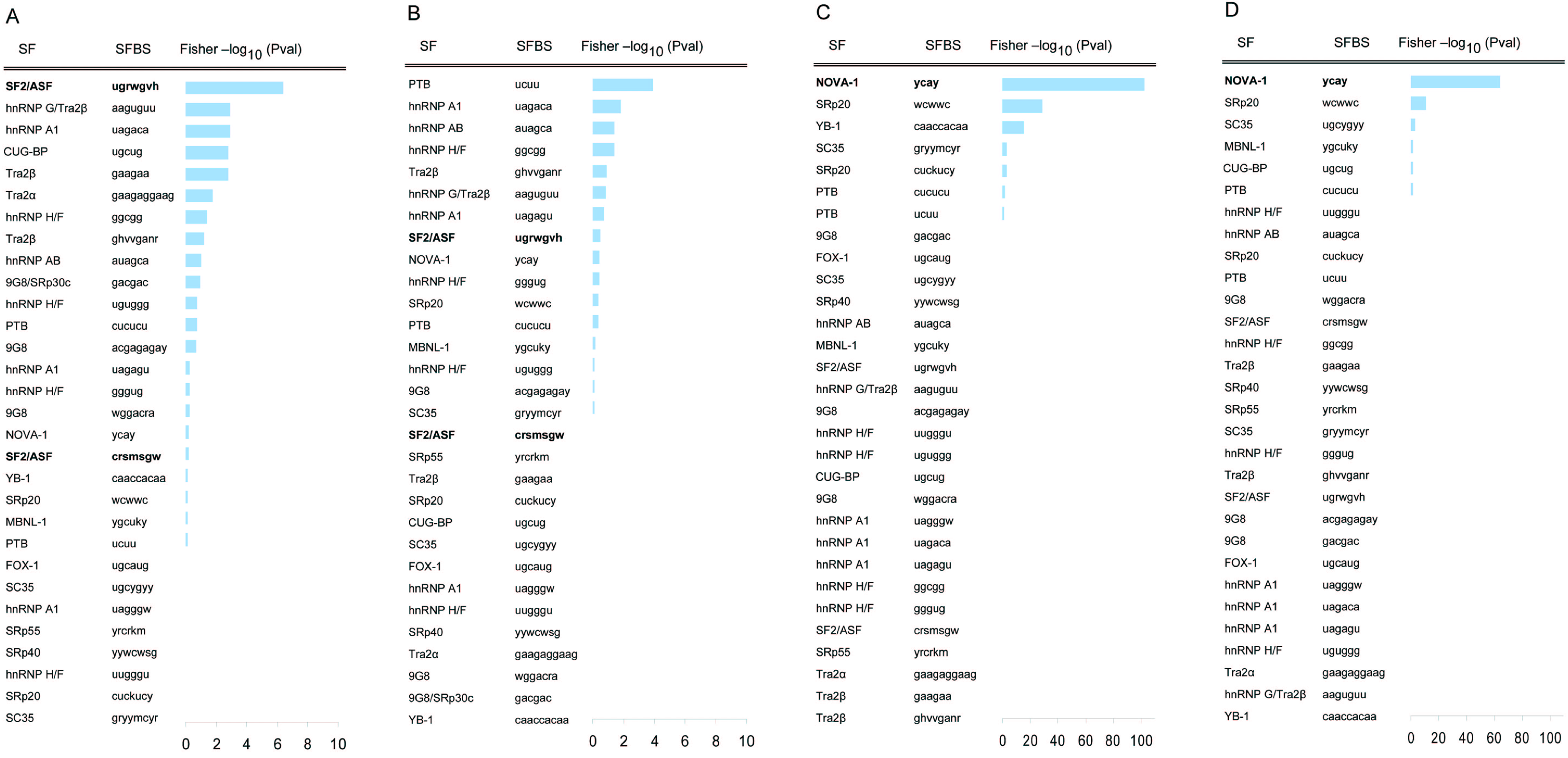

Figure S2: Analysis of the SF2/ASF and NOVA-1 CLIP datasets. The bar plots display *–log10*(*p*-value) for 30 different motifs when applying the Fisher Exact test to search for enrichment of scores above the threshold in the CLIP derived sequences compared to random sequences. (A, B) Results of the SF2/ASF dataset when using (A) COS(WR) and (B) Single Scores. (C, D) Results of the NOVA-1 dataset when using (C) COS(WR) and (D) Single Scores.
